# Behavioral Insights from Vaccine Adoption in Nigeria: Cross-Sectional Survey Findings

**DOI:** 10.2196/47817

**Published:** 2024-02-26

**Authors:** Sohail Agha, Ifeanyi Nsofor, Drew Bernard, Sarah Francis, Nandan Rao

**Affiliations:** 1 Behavior Design Lab Stanford University Stanford, CA United States; 2 Behavioral Insights Lab Seattle, WA United States; 3 Centre for Family Health Initiative Abuja Nigeria; 4 Virtual Labs Corvallis, OR United States

**Keywords:** behavioral insights, COVID-19, Nigeria, surveys, vaccination

## Abstract

**Background:**

To generate behavioral insights for the development of effective vaccination interventions, we need approaches that combine rapid and inexpensive survey data collection with instruments based on easy-to-use behavior models. This study demonstrates how an inexpensive digital survey helped identify the drivers of COVID-19 vaccination in Nigeria.

**Objective:**

This study aims to illustrate how behavioral insights can be generated through inexpensive digital surveys.

**Methods:**

We designed and conducted a cross-sectional survey with multistage sampling. Data were collected from Nigerians (aged ≥18 years) from 120 strata based on age, sex, state, and urban or rural location. Respondents were recruited via advertisements on Meta platforms (Facebook and Instagram) using the Virtual Lab open-source tool. We used a Meta Messenger chatbot for data collection; participants were compensated with 400 naira (US $0.87 cents). Data collection took 2 weeks. In total, 957 respondents completed the survey, at an advertising cost of US $1.55 per respondent. An 18-item instrument measuring core motivators, ability barriers, sociodemographic characteristics, and respondents’ vaccination status was pretested before data collection. We ran separate logistic regression models to examine the relationships between vaccine uptake and core motivators, ability barriers, and sociodemographic variables. A final model that predicted vaccine uptake included all 3 sets of variables.

**Results:**

About 56% (n=540) of respondents reported that they had received at least 1 COVID-19 vaccination. Three core motivators were positively associated with vaccine uptake: the belief that the COVID-19 vaccine promised a better life (adjusted odds ratio [aOR] 3.51, 95% CI 2.23-5.52), the belief that the vaccine would allow respondents to do more things they enjoyed (aOR 1.97, 95% CI 1.33-2.93), and respondents’ perception that their friends and family members accepted their decision to get vaccinated (aOR 1.62, 95% CI 1.06-2.48). Two ability barriers were negatively associated with vaccine uptake: cost- or income-related concerns lowered the odds of being vaccinated (aOR 0.35, 95% CI 0.24-0.50) and the lack of availability of vaccines at places respondents routinely visited also lowered their odds of being vaccinated (aOR 0.29, 95% CI 0.21-0.40). After adjusting for other variables, the perceived fear of getting COVID-19 and the hardship associated with the disease were no longer associated with vaccine uptake.

**Conclusions:**

These findings suggest that hope is more important for Nigerians than fear when it comes to vaccine adoption, enjoying life is more important than worrying about getting the disease, and approval from friends and family is more powerful than their disapproval. These findings suggest that emphasizing the benefits of leading a fuller life after being vaccinated is more likely to succeed than increasing Nigerians’ fear of COVID-19. This study identifies a very different set of factors associated with COVID-19 vaccine adoption than previous Nigerian studies.

## Introduction

### Background

Globally, the COVID-19 pandemic has highlighted the importance of behavioral insights for increasing the use of preventive behaviors such as wearing a mask, social distancing, and getting vaccinated. However, recent studies on COVID-19 vaccination emerging from Nigeria and other low- and middle-income countries (LMICs) have primarily focused on identifying gaps in knowledge, attitudes, and beliefs associated with vaccine hesitancy. A number of these studies recommend educating health care workers (HCWs) and members of the general population on vaccine safety and efficacy and assume that equipping people with factually correct information will allay their concerns, increase their perceived risk of acquiring COVID-19, and lead to higher rates of vaccine adoption [1,2].

Data from other Nigerian studies, however, raise questions regarding the strength of the relationship between risk perception and vaccine acceptance. A recent hospital-based study in southern Nigeria found that, while most HCWs perceived themselves at risk of COVID-19, only about half were willing to receive the COVID-19 vaccine [3]. Substantial gaps between risk perception and willingness to accept a COVID-19 vaccine have been observed in the general population in northern Nigeria as well [4]. A study that interviewed over 5000 respondents across all states in Nigeria found that COVID-19 was not perceived as a threat by most respondents [5].

What seems to be an important determinant of vaccine acceptability in Nigeria is trust in the vaccine manufacturing process, health system, government [6], and institutions involved in risk communication on behalf of the government [7]. Yet, despite multiple studies showing a weak relationship between risk perception and willingness to adopt a COVID-19 vaccine in Nigeria, researchers continue to recommend the provision of factually correct information to fill “information gaps” [4].

A recent systematic review of the COVID-19 literature in Nigeria shows that vaccination rates among those at high risk of COVID-19, such as HCWs, were lower than among those at low risk of COVID-19 [8]. Given the weak relationship between risk perception and vaccine uptake in Nigeria, it is not surprising that a recent evaluation found that risk communication efforts in Nigeria were inadequate in sustaining changes in behavior observed at the beginning of the pandemic [9]. Lawal [9] showed that during the first 30 days of the discovery of COVID-19 in Nigeria, and until the national lockdown, public interest in learning about the disease surged. Visits to public places such as grocery stores declined during this period as stringent government policies resulted in reduced mobility of the population. The study by Lawal [9] found that, as the Nigerian population started becoming aware of the disease, there was a slight decline in the number of COVID-19 cases. However, this decline occurred for a relatively short period of time. The number of new cases started increasing again as the initial effects of risk communication interventions dissipated. Lawal [9] concluded that Nigerians listened to messages telling them to take preventive measures such as social distancing or masking for some time but eventually got tired of the messages and stopped responding to them. In part, this was because the recommended public health precautions did not fit well in the context in which they lived their lives [9].

A clear picture of the drivers of COVID-19 vaccine hesitancy does not emerge from the recent public health literature on Nigeria, in part because much of this work is not based on a behavioral framework. The importance of theory-based work to understand the drivers of vaccine acceptance and design appropriate interventions has been emphasized [10]. In the absence of a clearly articulated framework for understanding vaccine-related behavior, it is difficult to interpret the findings of individual studies and arrive at a clear picture of what drives vaccine uptake in Nigeria.

As a result, there is very limited guidance available to support Nigerian practitioners in designing interventions that might accelerate COVID-19 vaccine uptake. For example, although many recent studies emphasize the importance of implementing health promotion interventions or increasing HCWs’ ability to communicate more effectively with members of the general public, most of these studies do not provide any guidance on what the content of this communication should be or what strategy should be used to persuade adults to get vaccinated. Thus, the available research is at a standstill in terms of providing insights that would help in designing more effective behavioral interventions to accelerate vaccine uptake in Nigeria.

An important reason that the literature does not provide a clear direction for the design of behavioral interventions is the lack of use of behavioral frameworks in explaining vaccine acceptance and uptake. Of the more than 20 peer-reviewed publications reviewed for this paper, we found only 1 that used a behavior model to interpret its findings [5]. This is not surprising as researchers have noted the limited use of behavioral frameworks in public health research and practice for over a decade [11-14].

The need for a behavior model that can be used to explain the vaccine adoption process in simple terms that resonate with practitioners is urgent. While a broader discussion of what a practitioner-friendly behavior model should comprise of is merited, the characteristics of such a model have been proposed [15]. A minimum criterion should be that the use of the model leads to deliberate programmatic decisions, a greater emphasis on strengthening activities supported by behavioral research findings, and the elimination of activities that are not evidence based.

The practitioner-friendly model used in this study, the Fogg Behavior Model (FBM), was introduced in the public health literature in 2019 to explain the effects of a social marketing behavior change campaign on the adoption of condoms by married men in Pakistan [13]. More recently, it has been used to identify behavioral drivers associated with the (1) adoption of COVID-19 vaccination by a low-income population in Cote d’Ivoire [16], (2) adoption of iron folate by pregnant women in India [15], (3) uptake of COVID-19 vaccination by HCWs in Nigeria [17,18], and (4) use of contraception by adolescent girls and young women in Nigeria [19]. A recent study also demonstrates the use of the FBM in making timely programmatic adjustments to a contraceptive social marketing intervention implemented in Nigeria [20]. To the best of our knowledge, this is the first time that the FBM is being applied to understand the dynamics of COVID-19 vaccine adoption in the general population of Nigeria.

### FBM: Motivation and Ability as the Drivers of Behavior

**Figure 1:**
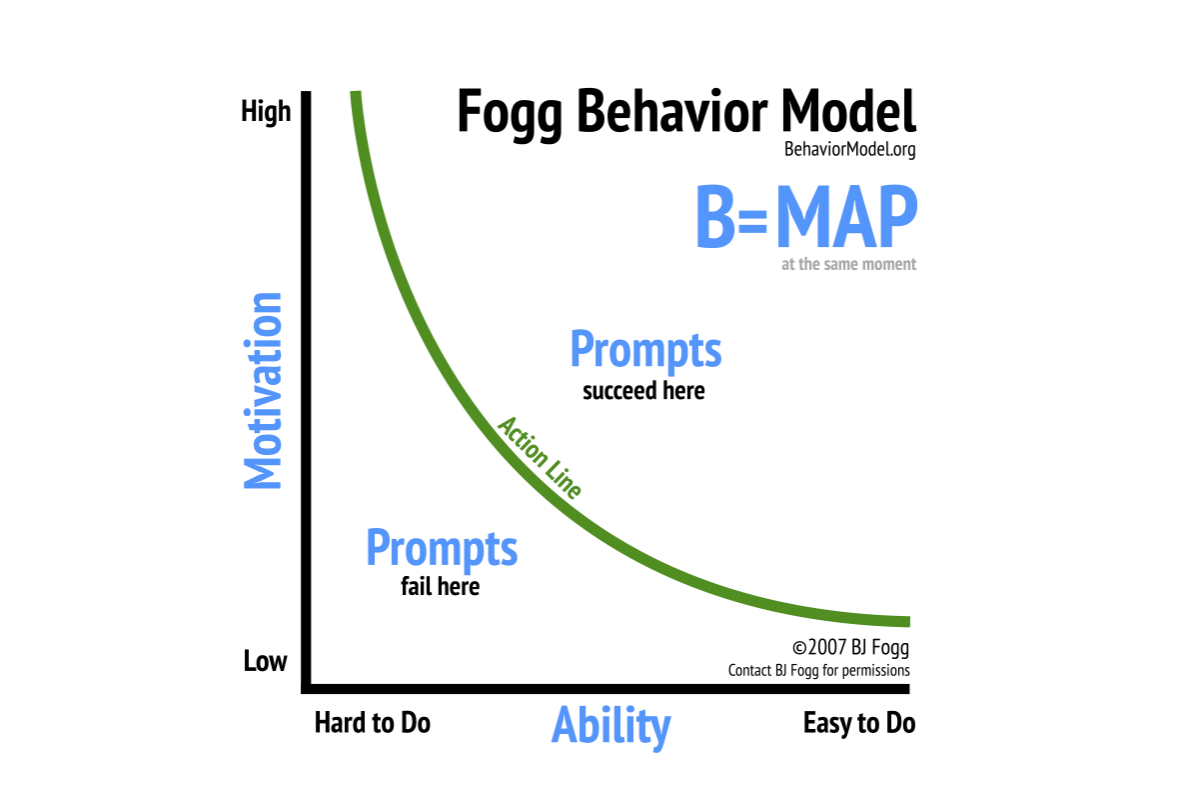
Fogg Behavior Model (reproduced from Fogg [22], with permission from BJ Fogg).

The FBM is a model developed for use by practitioners to understand the drivers of human behavior and assist them in the design of behavior change interventions. Fogg states that behavior happens when motivation, ability, and a prompt happen at the same moment. As shown in Figure 1, the model can be visualized in 2 dimensions. Figure 1 shows motivation along the y-axis and ability along the x-axis. Motivation ranges from high to low for any behavior. Ability also ranges from high to low for any behavior. For simplicity, we describe a behavior as being easy to do or hard to do. For a prompt to work, a person needs to have sufficient motivation and ability. The motivation-ability threshold is reflected by an action line in the FBM. Behavior occurs when a person whose motivation and ability are above the action line is prompted. The prompt does not work if the person does not have sufficient motivation to undertake the behavior and finds the behavior hard to do, that is, they are below the action line [21].

## Methods

### Questionnaire Design: Core Motivators

Fogg defines motivation as having 3 components: anticipation, sensation, and belonging. Anticipation reflects the hopes and fears a person associates with a behavior. Sensation reflects the pleasure or pain a person associates with a behavior. Belonging is reflected by the acceptance or rejection of the behavior by people whose opinions a person considers important.

We conducted a review of the literature to identify relevant constructs and appropriate measures of motivation and ability. The survey questionnaire (Multimedia Appendix 1) was designed using instruments developed to test motivation and ability constructs in the FBM. These instruments had been previously tested in the general population in Cote d’Ivoire and with HCWs in Nigeria and had shown acceptable levels of reliability [16,18]. Respondents were allowed to answer how strongly they agreed or disagreed with 6-point Likert scale items measuring core motivators, such as hope, fear, pleasure, pain, acceptance, and rejection, associated with the adoption of the COVID-19 vaccine.

### Ability Barriers

Fogg defines ability in terms of 5 barriers: time, money, the physical effort required to adopt a behavior, the mental effort required to adopt a behavior, and whether the behavior fits into the person’s routine [21]. Fogg initially considered social deviance (or social norms) as part of ability but did not include social norms as an ability barrier in later iterations of the model [22], possibly because of the complexity of the relationship between norms and behavior. To capture ability barriers, respondents could answer how strongly they agreed with items on a 5-point Likert scale. These items captured the 5 ability factors related to COVID-19 vaccine uptake: time, money, mental effort, physical effort, and routine. The instrument also contained questions on sociodemographic variables. In total, the instrument comprised 18 questionnaire items.

The instrument was pretested twice in Nigeria with samples of approximately 100 respondents. The first pretest showed that the relationship among variables measuring motivation, ability, and COVID-19 vaccine uptake was in the expected direction, with 1 exception. The variable measuring agreement or disagreement with the question “Many of my friends and family would think poorly of me if they knew I had taken the COVID-19 vaccine” did not demonstrate the expected relationship with vaccine uptake. This was replaced with the statement “Most people I know have obtained the COVID-19 vaccination.” The second pretest showed that the latter response was associated with the outcome in the expected direction. All other items included in the instrument demonstrated the expected relationships with vaccine uptake in both pretests.

### Survey Design and Sampling

We implemented a cross-sectional survey with multistage sampling. Nigeria is a large and diverse country with 36 states and the Federal Capital Territory of Abuja. These states and the Federal Capital Territory are grouped into 6 geopolitical zones: northwest, northeast, north central, southwest, southeast, and south-south. Although this survey was not designed to be a representative survey of Nigeria, we aimed to capture the diversity of the country’s population by ensuring that respondents from 1 state within each geopolitical zone were sampled. Thus, the states were selected at the first stage. Sokoto state was selected from the northwest, Bauchi state from the northeast, Niger state from north central, Lagos state from the southwest, Anambra state from the southeast, and Rivers state from the south-south. The state selected within each zone reflects its socioeconomic, religious, and ethnic diversity. In addition, these 6 states had ongoing COVID-19 vaccination campaigns to help ensure that survey findings would be useful for COVID-19 program managers in Nigeria.

The sample was stratified by 6 states, 5 age groups, male and female sex, and urban or rural location. This resulted in 120 strata from which respondents were sampled. We ran a total of 120 different ad sets targeting respondents based on the characteristics mentioned above.

Respondents were recruited via advertisements on the Meta digital ad platforms (Facebook and Instagram) using the Virtual Lab open-source tool [23]. The Virtual Lab tool ran ads targeting respondents in all 120 strata. We used a Meta Messenger chatbot for the survey data collection, compensating respondents who completed the survey with 400 naira (US $0.87 cents) in mobile phone credit. Respondents who clicked on the ads were directed to the messenger bot. Of the 214,335 male and female respondents who reached through the ads, 3660 clicked on the link, 1367 started the survey, and 1011 answered most survey questions.

Respondents could complete the survey in one go or start the survey, stop, and return to complete it later. In total, 957 respondents answered all questions in the survey at an advertising cost of US $1.55 per person. Data from these 957 respondents are used for the analysis.

The outcome of interest for this study was having received at least 1 COVID-19 vaccination. The Government of Nigeria’s data on the number of vaccinations provided in 2022 suggested that 50% of Nigerians had received at least 1 COVID-19 vaccination. A sample size calculation was made using an outcome value of 50% and a design effect of 1.5. We estimated that a sample size of 900 would provide a margin of error of 4 percentage points on the outcome of interest.

### Statistical Analysis: Relationships Among Core Motivators, Ability Barriers, and Vaccine Uptake

Univariate analysis was conducted to provide the frequency distributions of core motivators, ability barriers, and sociodemographic characteristics of the sample. Bivariate analysis was conducted to explore the relationships between core motivators, ability factors, sociodemographic characteristics, and having received at least 1 COVID-19 vaccination. Multivariate logistic regression analysis was conducted to identify which core motivators, ability factors, and sample characteristics had a significant relationship with vaccine uptake [24].

We also ran a final multivariate model to determine whether there was any change in the relationship between individual variables and vaccine uptake after taking the 3 sets of variables (core motivators, ability factors, and sociodemographic characteristics) into account. Adjusted odds ratios (aORs) from the analyses are shown in the tables. *P* values were considered statistically significant at *P*<.05.

### Ethical Considerations

The ethical approval for the study was obtained from Nigeria’s National Health Research Ethics Committee (NHREC/01/01/2007). Informed consent was obtained from all respondents to the quantitative survey. Respondents were assured that all written and recorded data would be kept confidential by using codes to identify participants instead of names or any other personal identifiers. Respondents were informed about their right to refuse to participate in the study or withdraw at any time during the interview.

## Results

### Core Motivators and COVID-19 Vaccine Uptake

Table 1 shows the frequency distributions of core elements of motivation identified by the FBM, cross-tabulations between core motivators and COVID-19 vaccine uptake and the aOR of COVID-19 vaccination. The first column of Table 1 shows that about three-fourths (n=726, 75.9%) of survey respondents agreed or strongly agreed with the statement that getting vaccinated against COVID-19 allows a person to live a better life, a measure of hope. Nearly half (n=444, 46.4%) of respondents reported that getting vaccinated protected them and their families from hardship, a measure of fear. Nearly two-thirds (n=610, 63.7%) of respondents agreed or strongly agreed with the statement that getting vaccinated allows a person to do the things they enjoy, a measure of pleasure. About 81.5% (n=780) reported that vaccination reduces the likelihood of getting or spreading COVID-19, a measure of pain. About 79.1% (n=757) of respondents reported that many of their family and friends approve of the COVID-19 vaccination, a variable measuring social acceptance. Consistent with the high social acceptance of the COVID-19 vaccination, rejection of the vaccine was much lower, that is, 37.1% (n=355) of respondents reported that most of their family and friends did not approve of the COVID-19 vaccination.

**Table 1:** Frequency distributions of core motivators, cross-tabulations, and the adjusted odds of COVID-19 vaccination in Nigeria.

Core motivators		Frequency distributions of core motivators (N=957, 100%), n (%)	Nigerian adults who obtained at least 1 vaccination (n=540, 56.4%), n (%)	*P* value		Adjusted odds of obtaining at least 1 COVID-19 vaccination, aOR (95% CI)^a^	
**Hope: Vaccination allows a person to live a better life**					<.001		
	Agree or strongly agree	726 (75.9)	483 (66.5)			3.40 (2.27-5.09)	
	Disagree or strongly disagree or do not know	231 (24.1)	57 (24.7)			1.00 (Reference)	
**Fear: Getting vaccinated protects people from hardship**					<.001		
	Agree or strongly agree	444 (46.4)	301 (67.8)			1.09 (0.78-1.51)	
	Disagree or strongly disagree or do not know	513 (53.6)	239 (46.6)			1.00 (Reference)	
**Pleasure: Getting vaccinated allows people to do more things they enjoy**					<.001		
	Agree or strongly agree	610 (63.7)	411 (67.4)			1.84 (1.30-2.62)	
	Disagree or strongly disagree or do not know	347 (36.3)	129 (37.2)				
**Pain: Getting vaccinated reduces the likelihood of getting or spreading COVID-19**					<.001		
	Agree or strongly agree	780 (81.5)	477 (61.2)			1.15 (0.76-1.73)	
	Disagree or strongly disagree or do not know	177 (18.5)	63 (35.6)			1.00 (Reference)	
**Acceptance: Many friends and family approve vaccination**					<.001		
	Agree or strongly agree	757 (79.1)	472 (62.4)			1.76 (1.21-2.58)	
	Disagree or strongly disagree or do not know	200 (20.9)	68 (34)			1.00 (Reference)	
**Rejection: Most family and friends do not approve of vaccination**					.56		
	Agree or strongly agree	355 (37.1)	344 (57.1)			1.06 (0.79-1.43)	
	Disagree or strongly disagree or do not know	602 (62.9)	196 (55.2)			1.00 (Reference)	

^a^Pseudo *R*^2^=11.85%

The second column of Table 1 shows cross-tabulations between the core motivators of vaccination and vaccine uptake. There were large differences in vaccine uptake by core motivators at the bivariate level. Respondents who associated a COVID-19 vaccination with the hope of a better life had a 42-percentage point higher rate of vaccination (n=483, 66.5% vs n=57, 24.7%; *P*<.001). Those who feared the hardship that COVID-19 infection would bring had a 21-percentage point higher vaccination rate than other respondents (n=301, 67.8% vs n=239, 46.6%; *P*<.001). The pleasure that respondents associated with being able to do what they enjoyed doing because of being vaccinated was reflected by a 30-percentage point higher rate of vaccination (n=411, 67.4% vs n=129, 37.2%; *P*<.001). Respondents’ concern that not being vaccinated would result in getting or spreading COVID-19 was associated with a 25-percentage point higher rate of vaccination (n=477, 61.2% vs n=63, 35.6%; *P*<.001). Acceptance of the vaccine by friends and family was associated with a 28-percentage point higher vaccination rate (n=472, 62.4% vs n=68, 34%; *P*<.001). It is interesting that social rejection, or the lack of approval of the vaccination by family members, was not associated with vaccine uptake.

The third column of Table 1 shows the adjusted odds of COVID-19 vaccination. With all core motivators in the model, Nigerians who believed that COVID-19 vaccination was associated with a better life, had 3 times higher odds of getting vaccinated (aOR 3.40, 95% CI 2.27-5.09). Those who believed that getting vaccinated would allow them to do more things that they enjoyed were more likely to be vaccinated (aOR 1.84, 95% CI 1.30-2.62). Friends and family members’ acceptance of their decision to get vaccinated was associated with a higher vaccination rate (aOR 1.76, 95% CI 1.21-2.58).

### Ability Barriers and COVID-19 Vaccine Uptake

Table 2 shows the frequency distributions of ability factors identified by the FBM, cross-tabulations between ability factors and COVID-19 vaccine uptake, and the adjusted odds of COVID-19 vaccination. The first column of Table 2 shows that about 54% (n=516) of respondents felt that their family or work responsibilities made it difficult for them to get vaccinated. This variable measures the constraint of time. Over a third (n=347, 36.3%) of respondents felt that the cost or loss of income associated with getting vaccinated was a barrier. Nearly 40% (n=382) of respondents felt that the physical effort required made it difficult to get vaccinated. Nearly half (n=437, 46%) of the respondents felt that the decision to get vaccinated was difficult. This variable measures the mental effort required to get vaccinated. About 42% (n=401) of respondents reported that not having the vaccine available in places they routinely visited was a barrier to getting vaccinated. The latter measures the routine associated with adopting a behavior.

**Table 2:** Frequency distributions of ability factors, cross-tabulations, and the adjusted odds of COVID-19 vaccination in Nigeria.

Ability			Frequency distributions of ability factors (N=957, 100%), n (%)		Nigerian adults who obtained at least one vaccination (n=540, 56.4%), n (%)		*P* value		Adjusted odds of obtaining at least one COVID-19 vaccination^a^
**Time: Family or work responsibilities make it difficult to find time**									
	Agree or strongly agree	516 (53.9)		277 (53.7)		.06		0.94 (0.69-1.27)	
	Disagree or strongly disagree or do not know	441 (46.1)		263 (59.6)				1.00 (Reference)	
**Money: Costs or loss of income make it difficult**									
	Agree or strongly agree	347 (36.3)		158 (45.5)		<.001		0.45 (0.33-0.62)	
	Disagree or strongly disagree or do not know	610 (63.7)		382 (62.6)				1.00 (Reference)	
**Physical effort: Physical effort makes it difficult**									
	Agree or strongly agree	382 (39.9)		218 (57.1)		.74		1.46 (1.06-2.01)	
	Disagree or strongly disagree or do not know	575 (60.1)		322 (56.0)				1.00 (Reference)	
**Mental effort: Decision to get vaccine is difficult**									
	Agree or strongly agree	437 (45.7)		237 (54.2)		.21		0.94 (0.70-1.27)	
	Disagree or strongly disagree or do not know	520 (54.3)		303 (58.3)				1.00 (Reference)	
**Routine: Vaccine not available where I routinely visit**									
	Agree or strongly agree	401 (41.9)		141 (35.2)		<.001		0.21 (0.16-0.28)	
	Disagree or strongly disagree or do not know	556 (58.1)		399 (71.8)				1.00 (Reference)	

^a^Pseudo *R*^2^=12%.

The second column of Table 2 shows cross-tabulations between ability factors and vaccine uptake. Respondents who agreed or strongly agreed with the statement that costs or loss of income were a barrier reported a 17-percentage point lower vaccination rate compared to others (n=158, 45.5% vs n=382, 62.6%; *P*<.001). The lack of availability of the COVID-19 vaccine in places that they routinely visited was associated with a 37-percentage point lower rate of vaccination (n=141, 35.2% vs n=399, 71.8%; *P*<.001).

The third column of Table 2 shows the adjusted odds of a COVID-19 vaccination. With all ability factors in the model, Nigerians who believed that the cost or the loss of income made it difficult to obtain a COVID-19 vaccination were less likely to get vaccinated (aOR 0.45, 95% CI 0.33-0.62). The lack of availability of vaccines at places respondents routinely visited was associated with a lower likelihood of vaccination (aOR 0.21, 95% CI 0.16-0.28). Contrary to our expectations, respondents who felt that physical effort makes it difficult to get vaccinated were more likely to be vaccinated (aOR 1.46, 95% CI 1.06-2.01).

### Sociodemographic Factors and COVID-19 Vaccine Uptake

Table 3 shows the frequency distributions of sociodemographic characteristics of respondents in the sample, cross-tabulations between these characteristics and vaccine uptake, and the adjusted odds of COVID-19 vaccination. The first column of Table 3 shows that, as expected from a digital survey, the sample had relatively young participants: 56% (n=531) of respondents were aged 18-29 years and 16% (n=151) were aged 40 years and older. Male participants represented a higher proportion of the sample (n=592, 61.9%). About 39% (n=371) of respondents had a primary or secondary school certificate, one quarter (n=231, 24.1%) had an ordinary national diploma (OND) or a higher national diploma (HND), and one-third of respondents (n=317, 33.4%) had a bachelor’s or higher degree. A majority of respondents were from urban areas: 57% (n=544) were from cities, 34% (n=323) from towns, and 9% (n=90) from rural areas.

**Table 3:** Frequency distributions of sociodemographic variables, cross-tabulations, and the adjusted odds of COVID-19 vaccination in Nigeria.

Demographic		Sample characteristics (N=957, 100%), n (%)	Nigerian adults who obtained at least 1 vaccination (n=540, 56.4%), n (%)	*P* value		Adjusted odds of obtaining at least 1 COVID-19 vaccination, aOR (95% CI)^a^	
**Age (years)**					.71		
	18-29	531 (55.5)	300 (56.5)			1.31 (0.89-1.93)	
	30-39	275 (28.7)	159 (57.8)			1.28 (0.85-1.93)	
	≥40	151 (15.8)	81 (53.8)			1.00 (Reference)	
**Sex**					.06		
	Male	592 (61.9)	348 (58.8)			1.00 (Reference)	
	Female	365 (38.1)	192 (52.6)			0.73 (0.56-0.95)	
**Education**					<.001		
	Primary or secondary school certificate	371 (38.8)	188 (50.7)			1.00 (Reference)	
	Ordinary national diploma (OND)	111 (11.6)	72 (64.9)			1.90 (1.22-2.98)	
	Higher national diploma (HND)	120 (12.5)	87 (72.5)			2.62 (1.64-4.19)	
	Bachelors or higher	317 (33.4)	169 (53.3)			1.12 (0.81-1.55)	
	Other	38 (4)	24 (63.2)			1.79 (0.89-3.60)	
**Location**					.02		
	City	544 (56.8)	320 (58.8)			1.00 (Reference)	
	Town	323 (33.8)	181 (56.0)			0.88 (0.68-1.17)	
	Rural	90 (9.4)	39 (43.3)			0.56 (0.35-0.89)	

^a^Pseudo *R*^2^=2.79%.

The second column of Table 3 shows cross-tabulations between sociodemographic characteristics and vaccine uptake. There was no statistically significant difference in the COVID-19 vaccination rate by age or sex. Education was associated with vaccine uptake: respondents with an OND (n=72, 64.9% vs n=188, 50.7%; *P*<.001) or an HND (n=87, 72.5% vs n=188 50.7%; *P*<.001) were more likely to be vaccinated than respondents with a primary or secondary school certificate. Urban residence was also associated with higher vaccine uptake: respondents from rural areas were significantly less likely to have obtained the COVID-19 vaccination (n=39, 43.3% vs n=320, 58.8%; *P*=.02).

The third column of Table 3 shows the adjusted odds of COVID-19 vaccination. With all sociodemographic characteristics in the model, female participants were less likely to get vaccinated (aOR 0.73, 95% CI 0.56-0.95). Having an OND (aOR 1.90, 95% CI 1.22-2.98) or a HND (aOR 2.62, 95% CI 1.64-4.19) increased a respondent’s likelihood of being vaccinated. Nigerians living in rural areas were less likely to be vaccinated (aOR 0.56, 95% CI 0.35-0.89).

### Full Model: Core Motivators, Ability Barriers, and Sociodemographic Characteristics

Table 4 shows the adjusted odds of COVID-19 vaccine uptake in Nigeria. The 3 core motivators identified earlier remained significant after adjusting for ability factors and sociodemographic characteristics. Nigerians who believed that the COVID-19 vaccination was associated with the promise of a better life were more likely to be vaccinated (aOR 3.51, 95% CI 2.23-5.52). Nigerians who felt that the vaccination would allow them to do more things they enjoyed were more likely to be vaccinated (aOR 1.97, 95% CI 1.33-2.93). Respondents’ friends’ and family members’ acceptance of their decision to get vaccinated was associated with a higher likelihood of their being vaccinated (aOR 1.62, 95% CI 1.06-2.48).

**Table 4:** Adjusted odds (aOR) of COVID-19 vaccination in Nigeria.

					Adjusted odds of obtaining at least 1 COVID-19 vaccination, aOR (95% CI)^a^
**Core motivators**					
	Getting vaccinated allows a person to live a better life (hope)			3.51 (2.23-5.52)	
	Getting vaccinated protects people from hardship (fear)			1.03 (0.71-1.49)	
	Allows people to do more things they enjoy (pleasure)			1.97 (1.33-2.93)	
	Reduces the likelihood of getting or spreading COVID-19 (pain)			1.17 (0.74-1.84)	
	Many friends and family approve vaccination (acceptance)			1.62 (1.06-2.48)	
	Most family and friends do not approve vaccination (rejection)			1.18 (0.83-1.67)	
**Ability factors**					
	Family or work responsibilities make it difficult (time)			0.85 (0.61-1.19)	
	Costs or loss of income make it difficult (money)			0.35 (0.24-0.50)	
	Physical effort makes it difficult (physical effort)			1.45 (1.02-2.07)	
	The decision to get the vaccine is difficult (mental effort)			1.08 (0.77-1.51)	
	Vaccine not available where I routinely visit (routine)			0.29 (0.21-0.40)	
**Sociodemographic factors**					
	**Age (years)**				
		18-29	1.09 (0.68-1.74)		
		30-39	1.14 (0.70-1.86)		
		≥40	1.00 (Reference)		
	**Sex**				
		Male	1.00 (Reference)		
		Female	0.80 (0.58-1.10)		
	**Education**				
		Primary or secondary school certificate	1.00 (Reference)		
		Ordinary national diploma (OND)	2.27 (1.34-3.84)		
		Higher national diploma (HND)	3.57 (2.04-6.24)		
		Bachelors or higher	1.26 (0.86-1.85)		
		Other	1.88 (0.82-4.33)		
	**Location**				
		City	1.00 (Reference)		
		Town	0.90 (0.64-1.26)		
		Rural	0.54 (0.31-0.92)		

^a^Pseudo *R*^2=^23.51%.

The relationships between ability factors and vaccine uptake remained important after adjusting for sociodemographic characteristics and core motivators. Nigerians with cost- or income-related concerns were less likely to obtain a COVID-19 vaccination (aOR 0.35, 95% CI 0.24-0.50). The lack of availability of vaccines at places they routinely visited made them less likely to get vaccinated (aOR 0.29, 95% CI 0.21-0.40).

After adjusting for motivation and ability, female participants were no longer less likely to obtain a COVID-19 vaccination. Respondents with an OND (aOR 2.27, 95% CI 1.34-3.84) or HND (aOR 3.57, 95% CI 2.04-6.24) were more likely to be vaccinated than those with primary or secondary school certificates. Rural residents were less likely to be vaccinated than residents living in cities (aOR 0.54, 95% CI 0.31-0.92).

## Discussion

### Principal Findings

The findings of this study show that 56.4% (n=540) of Nigerian adults who responded to the digital survey had obtained at least 1 COVID-19 vaccination by October 2022. Several core motivators were associated with vaccine uptake, after adjusting for ability factors and sociodemographic variables. The beliefs that COVID-19 vaccination allows a person to live a better life and that it allows them to do more things that they enjoy increases the likelihood of being vaccinated. The belief that many friends and family members approve of COVID-19 vaccination is also associated with a greater likelihood of being vaccinated. Several ability barriers were also correlated with vaccine uptake, after adjusting for other variables. Respondents who felt that costs or loss of income associated with getting vaccinated made it difficult to get vaccinated were less likely to get vaccinated. The lack of availability of the COVID-19 vaccine at places respondents routinely visited was also negatively associated with vaccine uptake. The study also found a higher likelihood of Nigerians with OND or HND being vaccinated compared with those with primary or secondary school certificates and a lower likelihood of being vaccinated among rural respondents.

### Strengths and Limitations

While this study provides useful insights for program design, its limitations should be acknowledged. The first limitation of this study is that no causal inferences can be made from it because of its cross-sectional design. This design limitation may explain an unexpected study finding: after adjusting for other variables, the physical effort required to get vaccinated was associated with a higher rate of vaccination. This finding may reflect reverse causality; those who are vaccinated may be more aware of the physical effort required to obtain a COVID-19 vaccination. Further investigation is needed to determine whether the positive relationship between the perceived physical effort required to get vaccinated and receiving a vaccination holds only for those who have been vaccinated or for the full sample.

The second limitation of this study is that variables that were not measured may be responsible for the observed relationships. For example, while the relationship between the belief that vaccination allows a person to enjoy life more and vaccine uptake is powerful, there is a possibility that unmeasured factors are driving this relationship. Thus, developing messages around how vaccination can help a person lead a fuller life and testing them through relatively inexpensive digital campaigns would be important prior to implementing an at-scale campaign that focuses on this message.

The third limitation of the study is that it is not representative of all Nigerians in the 6 states in which it was conducted. This is reflected in the higher educational status of the survey sample: about 33% (n=317) of respondents had a bachelor’s or higher education. The participants were also relatively young; about 56% (n=531) of respondents were between 18 and 29 years. Moreover, male participants comprised a higher proportion of the sample than female participants. These findings are not uncommon for digital surveys conducted in LMICs.

A strength of this study is its cost efficiency and the timeliness with which the survey was conducted compared to face-to-face household surveys. A major barrier to the use of behavioral insights by practitioners in LMICs is the cost of data collection. Behavioral research is not well-funded in LMICs. Inexpensive digital surveys could substantially increase the ability of practitioners in LMICs to use behavioral insights to develop interventions that increase vaccine uptake.

### Future Directions

We do not know the extent to which the findings from surveys conducted by recruiting respondents through web-based advertising are comparable to the findings from population-based household surveys. Although some studies show broadly similar patterns between digital and population-based surveys [25], more research is needed to identify what types of systematic differences may exist between these 2 survey modalities. It is important, for example, to learn whether inferences from digital surveys apply to the behavior of individuals who are not on digital platforms.

Given our inability to generalize these findings beyond Nigerians who are on Facebook and Instagram, how can the findings of this survey be used? First, the findings may be used to design interventions on digital platforms as well as to evaluate the effectiveness of those interventions. Digital behavior change interventions may be evaluated by experimental studies on digital platforms that compare vaccine uptake between intervention and control groups. A growing proportion of the Nigerian population is now on Facebook and Instagram: between 31 million and 36 million Nigerians 13 and older use Facebook and Instagram each month. High exposure to messages that associate the COVID-19 vaccination with a better, more fulfilling life is achievable through advertising on digital platforms and at a fraction of the cost of advertising on traditional mass media channels such as television.

Future interventions could build upon the findings of this study by conducting qualitative research to determine which motivation or ability factors are relevant in locations where interventions are planned. A survey conducted in the most densely populated, low-income commune in Yugpognon, Cote d’Ivoire, using the FBM found broadly comparable findings: motivation and ability were powerful drivers of vaccine adoption, although the specific elements of motivation and ability that were relevant in Yugpognon were, not surprisingly, different [16].

Our findings raise several questions that should be answered through additional research. Answers to these questions may help in the design of more effective COVID-19 service delivery interventions. It would be useful, for example, to learn whether some of the places routinely visited by Nigerians are amenable to serving as COVID-19 vaccine delivery sites. Are such potential vaccine delivery sites likely to vary by age, sex, by urban or rural residence and are they suitable for cost-efficient provision of COVID-19 vaccinations?

Our sample consisted primarily of Nigerians living in cities and towns, with a minority of respondents living in rural areas. A larger proportion of rural respondents may be obtained from digital surveys that oversample rural areas. This may be done by capping the number of respondents from urban areas and allowing more time for responses to come in from rural areas. This would, however, have implications for the cost of the rural component of the survey.

### Comparison to Prior Work

Overall, the findings of our study provide a very different perspective on vaccine adoption in Nigeria than what is available in the peer-reviewed literature. Recent Nigerian studies on COVID-19 vaccine uptake have primarily focused on identifying gaps in knowledge, attitudes, and beliefs associated with vaccine hesitancy [1-3]. These studies place emphasis on the perceived risk of disease as a driver of vaccine uptake, despite mixed evidence on the role of risk perception on vaccine uptake in Nigeria [5,6,9]. Several of these recent studies propose that equipping people with factually correct information will allay their concerns, increase their perceived risk of acquiring COVID-19, and lead to higher rates of vaccine adoption. By contrast, a small but rapidly growing body of work is putting vaccine adoption in a behavioral context [13-19,26]. These studies take motivation and ability for behavior change into account in explaining the range of barriers that influence immunization decisions and suggest how programs should help individuals overcome them.

### Conclusions

These findings help us consider a very different approach to intervention design—one that builds upon what people want for their future, what gives them pleasure, and how they are influenced by the approval of their friends and family members. Our findings suggest that hope is more important for Nigerians than fear when it comes to vaccine adoption, social approval is more powerful than social disapproval, and enjoying life is more important than worrying about getting the disease.

These findings suggest that an approach that is based on increasing the perception of hope and pleasure associated with vaccine adoption as well as increasing network members’ social approval is likely to increase COVID-19 vaccine adoption in Nigeria. Our analysis also suggests that financial considerations play an important role in the uptake of COVID-19 vaccination in Nigeria. The costs associated with reaching a vaccination site or the loss of income associated with being away from work are important determinants of vaccine adoption. Nigerians with limited flexibility at work may find it challenging to visit a vaccination site during the hours that it is open. Consistent with this finding, making COVID-19 vaccines available at places that Nigerians visit routinely may have a large impact on vaccine uptake.

The use of a behavior model to understand drivers of COVID-19 vaccine uptake in Nigeria has helped provide a different perspective on vaccine-related decision-making in Nigeria than what is currently available in the published literature. The FBM, a model of human behavior rather than a model of health behavior per se, considers a broad range of factors influencing motivation, including an individual’s hopes and fears, the sensation of pleasure or pain that they get from a particular behavior, and the social influences on them associated with their identity. The model also measures ability constraints including bandwidth-related constraints such as time or cognitive constraints, financial constraints, physical effort–related constraints, and habits or routine-related constraints. By comparison to behavior models that focus on perceived risk of and susceptibility to disease, the FBM situates behavior within the broader context of a person’s life.

## Appendix

Multimedia Appendix 1 Survey questionnaire.

## Abbreviations

aOR: adjusted odds ratio
FBM: Fogg Behavior Model
HCW: health care worker
HND: higher national diploma
LMICs: low- and middle-income countries
OND: ordinary national diploma

## Data Availability

The data sets generated and analyzed during this study are available from the corresponding author on reasonable request.
