# Supplementary material for: Behavioral Insights from Vaccine Adoption in Nigeria: Cross-Sectional Survey Findings
Source: Interact J Med Res. 2024 Feb 26;13:e47817. doi: 10.2196/47817 (PMC10928521; doi:10.2196/47817)
Supplement: Multimedia Appendix 1 [file ijmr_v13i1e47817_app1.docx]

**Behavioral Drivers of COVID-19 Vaccine Uptake Survey Instrument**

September and October 2022

**Delivery Mechanism:** Targeted Facebook Ads

**Collection Method:** Facebook Messenger

**Compensation Offered:** 400 Naira in mobile credits

NOTE: Each Box below reflects a message that was sent and responded to by participants.

| **Section A: Concent** | | |
| --- | --- | --- |
| Constent 1. | Thank you!  We hope you will take part in this survey. It is designed to identify the key obstacles to getting the COVID-19 vaccination in Nigeria.  Your voice and views are very important. Your participation really does matter. Insights from this survey will be used to protect the health and wellbeing of all Nigerians.  If you qualify for and complete the survey, you will get 400 Naira in mobile credit. | OK |
| Consent 2. | We know you are busy and we have worked hard to keep the survey as short as possible. It will only take approximately 5 minutes to complete. Your participation is completely voluntary, and your responses will be kept confidential. This study has been approved by NHREC (see below for approval number and duration).  - NHREC Approval Number NHREC/01/01/2007  - Duration of Approval: 22/06/2022 to 21/06/2023 | OK |
| Consent 3. | We will never use or release any information connecting your answers with your personal identity. All of the answers that you and others provide, will be kept private — only the academic researchers will have access to this information. You can choose not to answer any question that you would prefer not to answer. | Continue |
| Consent 4. | Would you be willing to take the survey? | Yes  No |
| Consent 5. | Great, thank you! | Continue |

| **Section B. Motivation Questions:** | | |
| --- | --- | --- |
| Motivation 1.  **Global Question** | Getting vaccinated against COVID-19 was important to me. | A. Strongly agree  B. Agree  C. Disagree  D. Strongly disagree  E. Don't know/Can't say |
| Motivation 2.  **Hope** | Getting vaccinated against COVID-19 allows me to do more of the things I enjoy. | A. Strongly agree  B. Agree  C. Disagree  D. Strongly disagree  E. Don't know/Can't say |
| Motivation 3.  **Fear** | Getting vaccinated against COVID-19 helps protect me and my family from hardship. | A. Strongly agree  B. Agree  C. Disagree  D. Strongly disagree  E. Don't know/Can't say |
| Motivation 4.  **Pleasure** | Getting vaccinated against COVID-19 allows me to do more of the things I enjoy. | A. Strongly agree  B. Agree  C. Disagree  D. Strongly disagree  E. Don't know/Can't say |
| Motivation 5.  **Pain** | I worried about getting or spreading a serious illness if I was not vaccinated against COVID-19. | A. Strongly agree  B. Agree  C. Disagree  D. Strongly disagree  E. Don't know/Can't say |
| Motivation 6.  **Acceptance** | Many of my family and friends approved of me getting the COVID-19 vaccine. | A. Strongly agree  B. Agree  C. Disagree  D. Strongly disagree  E. Don't know/Can't say |
| Motivation 7.  **Rejection** | Many of my family and friends would think poorly of me if they knew I had taken the COVID-19 vaccine. | A. Strongly agree  B. Agree  C. Disagree  D. Strongly disagree  E. Don't know/Can't say |

| **Section 3. Ability** *(all items on a 5-point agreement scale)* | | |
| --- | --- | --- |
| Ability 1.  **Global Question** | Was it easy or difficult for you to get the COVID-19 vaccination? | A. Very difficult  B. A bit difficult  C. Quite easy  D. Very easy  E. Don’t know/Can’t say |
| Ability 2.  **Time** | My family and work responsibilities made it difficult for me to find time to get a COVID-19 vaccine. | A. Strongly agree  B. Agree  C. Disagree  D. Strongly disagree  E. Don't know/Can't say |
| Ability 3.  **Money** | ma | A. Strongly agree  B. Agree  C. Disagree  D. Strongly disagree  E. Don't know/Can't say |
| Ability 4.  **Physical effort** | The physical effort of getting the COVID-19 vaccine made it difficult. | A. Strongly agree  B. Agree  C. Disagree  D. Strongly disagree  E. Don't know/Can't say |
| Ability 5.  **Mental effort** | The decision to get the COVID-19 vaccine was difficult. | A. Strongly agree  B. Agree  C. Disagree  D. Strongly disagree  E. Don't know/Can't say |
| Ability 6.  **Social norms** | Most of family and friends do not approve of people getting vaccinated for COVID-19. | A. Strongly agree  B. Agree  C. Disagree  D. Strongly disagree  E. Don't know/Can't say |
| Ability 7.  **Routine** | The vaccine was available in places that I routinely visit. | A. Strongly agree  B. Agree  C. Disagree  D. Strongly disagree  E. Don't know/Can't say |

| **Section 4: Respondent Profile** | | |
| --- | --- | --- |
| Gender | What is your gender? | 1. Woman 2. Man 3. Non-binary or transgender   D. Prefer not to say |
| Age | How old are you? | A. 18-29 years  B. 30-39 years  C. 40-49 years  D. 50-59 years  E. 60+ years |
| Education | What is the highest educational level or degree that you have attained?  **SELECT ONE ONLY.** | -A. No formal education  -B. Primary School Certificate  -C. SSCE, GCE  -D. OND  -E. HND  -F. BSc  -G. MSc  -H. PhD  -I. Other (please specify) |
| Location Typle | 22. Do you live in a city, in a town, or in a rural area? | -A. City  -B. Town  -C. Rural area |

| **Section 5: Thank You** | | |
| --- | --- | --- |
|  | **— Thank you very much!**  Your participation provides valuable insight into the key obstacles to getting the COVID-19 vaccination in Nigeria.  You are eligible to receive 400 Naira in mobile credit. We will send the credit to your mobile. | - Continue |
|  | Please write a mobile number where we can send mobile credit:  {"validate": { "country": "NGA", "mobile": true }} | (xxx) xxx-xxx   - OK |
|  | Which mobile operator is associated with that number? | 1. 9Mobile (Etisalat) Nigeria 2. Airtel Nigeria 3. Glo Nigeria 4. MTN Nigeria |
|  | Thank you. You should receive the credit shortly. We will write to you again when the payment has been accepted by your mobile operator. |  |
